# The complete mitochondrial genome of common terrestrial frog (*Rana temporaria*)

**DOI:** 10.1080/23802359.2018.1507649

**Published:** 2018-08-29

**Authors:** Jun-Jie Chen

**Affiliations:** College of Animal Science and Veterinary Medicine, Henan Institute of Science and Technology, Xinxiang, China

**Keywords:** *Rana temporaria*, European common frog, mitogenome

## Abstract

In this study, we presented the complete mitochondrial genome of common terrestrial frog (*Rana temporaria*). The circular genome is 16,061 bp in length and contains 13 protein-coding genes (PCGs), 22 transfer RNA (tRNA) genes, 2 ribosome RNA genes and 1 D-loop control regions. The overall nucleotide composition is: 27.3% A, 28.8% T, 28.9% C, and 15.1% G, with a total G + C content of 44.0%. The phylogenetic tree was constructed to validate the taxonomic status of *Rana temporaria*, exhibiting it a well-supported monophyletic branch in the group of genus *Rana*.

The common frog (*Rana temporaria*), also known as the European common frog, is a semi-aquatic amphibian of the family Ranidae, found throughout much of Europe.

Common frogs can survive in very cold environments and can be found at high altitudes; the highest recorded are in the French and Swiss Alps at ∼2,630 m (Dolmen et al. 1997). And they are considered as a good model system for examining postglacial colonization as it is widespread throughout Europe (Teacher et al. 2009). In the present study, the complete mitogenome sequence of *R. temporaria* has been determined. The work provides the reference sequence of mitogenome for *R. temporaria* that may be utilized for further phylogenetic studies in the future.

The specimen was collected from Ammarnäs, Sweden (65°58′N, 16°12′E). The muscle tissue and total genomic DNA that was extracted through Animal Tissues Genomic DNA Extraction Kit (Solarbio, BJ, CN) were stored in the sequencing company (BGI Tech, Shenzhen, China). Total genomic DNA of muscle tissue was extracted through Animal Tissues Genomic DNA Extraction Kit (Solarbio, BJ, CN). Purified DNA was fragmented and used to construct the sequencing libraries following the instructions of NEBNext^®^ Ultra™ II DNA Library Prep Kit (NEB, BJ, CN). Whole genomic sequencing was performed by the Illumina HiSeq 2500 Sequencing Platform (Illumina, CA, USA). Adapters and low-quality reads were removed using the NGS QC Toolkit (Patel and Jain 2012). The mitochondrial reads from pre-filtered reads were screened out by bowtie2 (Langmead and Salzberg 2012) using other *Rana* mitochondrial genomes as references. Then assembly as implemented by SPAdes 3.9.0 (Bankevich et al. 2012). Gaps among contigs were filled by using MITObim V1.9 (Hahn et al. 2013). The determined genome was annotated using the MFannot tool (http://megasun.bch.umontreal.ca/cgi-bin/mfannot/mfannotInterface.pl), combined with manual corrections. tRNAs were annotated by ARWEN Web Server (Laslett and Canbäck 2008).

The complete mitogenome of *R. temporaria* (GenBank accession: MH536744) is a closed-circular molecule of 16,061 bp in length, which is well within the size range observed in the completely sequenced other *Rana* mitogenomes. It presents the typical set of 37 genes observed in metazoan mitogenomes, including 13 PCGs (*cox*1–3, *co*b, *nad*1–6, *nad*4L, *atp*6 and *atp*8), 22 tRNA genes, 2 genes for ribosomal RNA subunits (*rrn*S and *rrn*L) and 1 D-loop control regions. The overall nucleotide composition is: 27.3% A, 28.8% T, 28.9% C, and 15.1% G, with a total G + C content of 44.0%.

To validate the phylogenetic position of *R. temporaria*, the genome-wide alignment of 24 Ranoidea mitochondrial genomes (22 species in genus *Rana*; *Paa spinosa* and *Tomopterna cryptotis* were set as outgroup) was constructed by HomBlocks (Bi et al. 2018), resulting in 12,211 characters of each species, which including almost all PCGs and rRNA genes. The JMODELTEST 2.0.2 (Darriba et al. 2012) was used to ascertain the best-ft model of nucleotide substitution for sequences with Bayesian information criterion (BIC). Bayesian phylogenetic analysis was conducted with MrBayes 3.2.5 (Ronquist et al. 2012) based on the most appropriate GTR + G + I model. Four Markov chains were run for 10,000,000 generations to estimate the posterior probability (PP) distribution (sampling 1 tree with 100 replicates for each run). After discarding the first 2000 trees as burn-in that was referred to log likehood values, 50% majority-rule consensus trees were estimated for the remaining trees. As shown in the phylogenetic tree (Figure 1), the mt genome reported here exhibited a well-supported monophyletic branch in the group of genus *Rana*.

**Figure 1:**
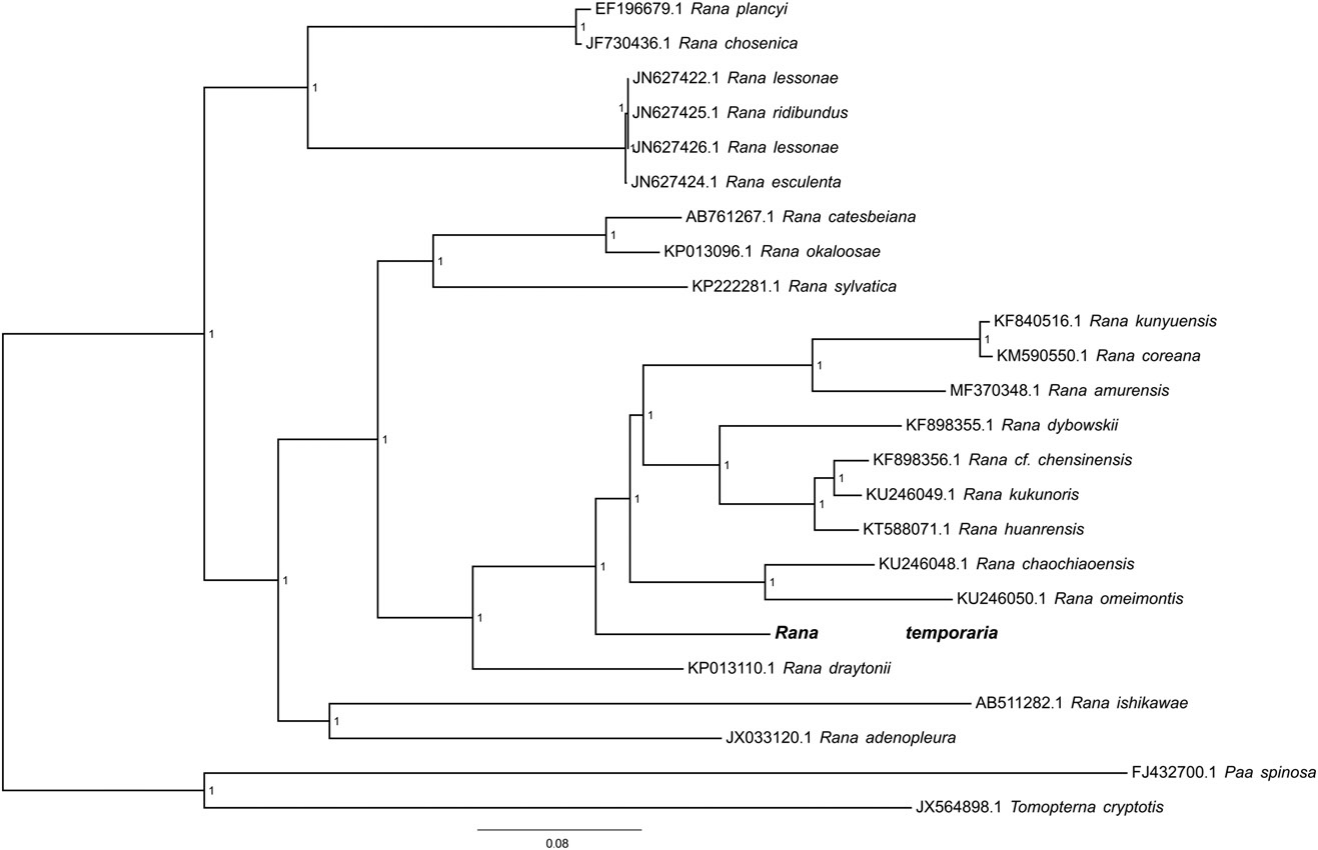
Bayesian phylogeny tree of relationships among 24 Ranoidea mitogenomes through whole genome-wide alignments constructed by HomBlocks. Bayesian posterior probability values (PP) are shown besides corresponding nodes.
